# In Vitro Susceptibility of Methicillin-Resistant Staphylococcus aureus to Ceftaroline: A Prospective Observational Study From a Tertiary Care Hospital in North India

**DOI:** 10.7759/cureus.110593

**Published:** 2026-06-10

**Authors:** Gaurav Jain, Ankit Agarwal, Balram Omar, Vanya Singh, Pooja Bhardwaj, Minakshi Singh, Sandhiya P Palanisamy, Anshima Agarwal

**Affiliations:** 1 Anesthesiology and Critical Care Medicine, All India Institute of Medical Sciences, Rishikesh, Rishikesh, IND; 2 Microbiology, All India Institute of Medical Sciences, Rishikesh, Rishikesh, IND; 3 Microbiology, Himalayan Institute of Medical Sciences, Swami Rama Himalayan University, Dehradun, IND

**Keywords:** antimicrobial susceptibility, ceftaroline, cephalosporin, e-test, methicillin-resistant staphylococcus aureus

## Abstract

Background

Methicillin-resistant *Staphylococcus aureus* (MRSA) continues to pose a major global public health concern due to its resistance to commonly used antibiotics. Ceftaroline has emerged as a promising therapeutic agent against MRSA; however, local susceptibility data remain limited. This study evaluated the in vitro susceptibility of clinical MRSA isolates to ceftaroline using the E-test strip method and described antimicrobial susceptibility patterns and clinical characteristics of affected patients in a tertiary care setting.

Methods

This prospective, observational, cross-sectional study included non-repetitive MRSA-positive clinical samples obtained from patients aged ≥1 year over a two-year period. Identification and baseline antimicrobial susceptibility testing were performed using the VITEK 2 automated microbiological identification system. Ceftaroline susceptibility was determined using E-test minimum inhibitory concentration (MIC) strips.

Results

Out of 180 patients assessed, 167 MRSA isolates were included in the final analysis. The mean patient age was 34.51 ± 21.35 years, with 103 (61.7%) males. Surgical departments accounted for 99 (59.3%) of isolates, while most patients were admitted to wards (134; 80.2%). Surgical site infections (61; 36.5%) and skin and soft tissue infections (30; 18.0%) were the most frequent diagnoses. Pus samples constituted 90 (53.9%) of the isolates. Ceftaroline demonstrated high in vitro activity against MRSA, with 166 (99.4%) of isolates being susceptible and only one (0.6%) resistant. Most MIC values ranged from 0.1 to 0.4 µg/mL. Tetracycline, vancomycin, linezolid, and daptomycin also demonstrated sensitivity rates above 80%.

Conclusions

These findings demonstrate excellent in vitro susceptibility of MRSA isolates to ceftaroline and support further multicenter studies incorporating clinical outcome data to evaluate its therapeutic role in tertiary care settings. Its clinical use should, however, be reserved for MRSA infections resistant to other antimicrobials.

## Introduction

Methicillin-resistant *Staphylococcus aureus* (MRSA) remains a major global public health concern because of its resistance to multiple commonly used antibiotics and its association with significant morbidity and mortality worldwide [1]. Despite ongoing infection control efforts, MRSA continues to cause both hospital-acquired and community-acquired infections, thereby complicating clinical management [2].

Resistance to β-lactam antibiotics in MRSA occurs primarily due to the acquisition of the mecA gene, which encodes an altered penicillin-binding protein (PBP2a) with reduced affinity for β-lactam antibiotics [3]. This mechanism renders MRSA resistant to most penicillins and cephalosporins that were previously considered first-line therapies for staphylococcal infections. Consequently, treatment often relies on alternative agents such as vancomycin, daptomycin, and linezolid [4]. However, these agents have several limitations, including slower bactericidal activity, suboptimal tissue penetration, adverse effects such as nephrotoxicity and myelosuppression, and the potential for emerging resistance [5].

Ceftaroline, a fifth-generation cephalosporin, has demonstrated activity against MRSA due to its strong affinity for PBP2a, enabling it to overcome the methicillin resistance mechanism [6]. Clinical studies have reported favorable outcomes with ceftaroline in the treatment of complicated skin and soft tissue infections and community-acquired pneumonia, including infections caused by MRSA [7]. However, the empirical use of ceftaroline requires reliable local antimicrobial susceptibility data.

In India, data regarding ceftaroline susceptibility among MRSA isolates remains limited [8]. Therefore, the present prospective observational study aimed primarily to evaluate the in vitro susceptibility of clinical MRSA isolates to ceftaroline using the E-test method in a tertiary care hospital. The secondary objectives were to determine the proportion of ceftaroline-susceptible isolates, describe antimicrobial susceptibility patterns among MRSA isolates, and characterize the clinical and demographic profiles of affected patients.

## Materials and methods

After obtaining institutional ethical approval and written informed consent, non-repetitive, consecutive clinical samples that were confirmed to be MRSA-positive, obtained from patients aged ≥1 year and diagnosed with local or systemic infections, were included in a prospective, observational, cross-sectional study between January 2024 and December 2025. Those who declined consent were excluded.

Clinical specimens were cultured on appropriate media, including 5% sheep blood agar and MacConkey agar, and incubated aerobically at 37°C for 18-24 hours. Colonies suggestive of *S. aureus* were identified based on colony morphology, Gram staining, and standard biochemical tests such as catalase and coagulase tests. Identification and baseline antimicrobial susceptibility testing were performed using the VITEK 2 Compact 60 automated microbiological identification system (bioMérieux, Marcy-l’Étoile, France) (GP ID card, AST-N628, ATCC 25923) by preparing a bacterial suspension equivalent to a 0.5 McFarland standard using the VITEK DENSICHEK device (bioMérieux; McFarland Reference, SN-DB08387), adhering to standard quality control measures. Methicillin resistance was determined based on resistance to cefoxitin and/or oxacillin according to standard guidelines [9], and isolates confirmed as MRSA were included in the study.

The in vitro susceptibility to ceftaroline was determined using the E-test minimum inhibitory concentration (MIC) method (bioMérieux). A fresh bacterial suspension was prepared from pure overnight colonies grown on blood agar plates and adjusted to a turbidity equivalent to a 0.5 McFarland standard, corresponding to approximately 1.5 × 10⁸ CFU/mL. Using a sterile cotton swab, the standardized bacterial suspension was evenly inoculated onto the surface of Mueller-Hinton agar plates to obtain a uniform lawn culture. After allowing the inoculated plates to dry for several minutes at room temperature, a ceftaroline E-test MIC strip containing a predefined antibiotic concentration gradient was aseptically placed on the agar surface using sterile forceps. The plates were then incubated aerobically at 35 ± 2°C for 18-24 hours. Following incubation, an elliptical zone of inhibition formed around the strip as a result of antibiotic diffusion through the agar medium. The MIC value for ceftaroline was determined by reading the point at which the ellipse of bacterial growth inhibition intersected the scale on the E-test strip. The obtained MIC values were interpreted according to standard guidelines, categorizing the isolate as susceptible (≤1 µg/mL), intermediate (2-4 µg/mL), or resistant (≥8 µg/mL) [9].

The primary outcome variable was the proportion of clinical MRSA isolates showing in vitro ceftaroline susceptibility using the E-test strip method. The secondary outcome variables included overall antimicrobial susceptibility trends of MRSA isolates, baseline patient characteristics, diagnoses, and risk factors.

The sample size was calculated using the OpenEpi Collection of Epidemiologic Calculator (version 3.01), assuming a 95% confidence level and an expected susceptibility of 95% to ceftaroline among MRSA, based on previous studies [3], with 5% absolute precision. This yielded a required sample size of 73. However, to enhance the study’s impact, all samples meeting the inclusion criteria during the study period were included. Statistical analyses were performed using IBM SPSS Statistics for Windows, version 23.0 (released 2015; IBM Corp., Armonk, NY, USA). Continuous variables were expressed as mean ± SD, while categorical variables were reported as frequencies and percentages.

## Results

**Figure 1:**
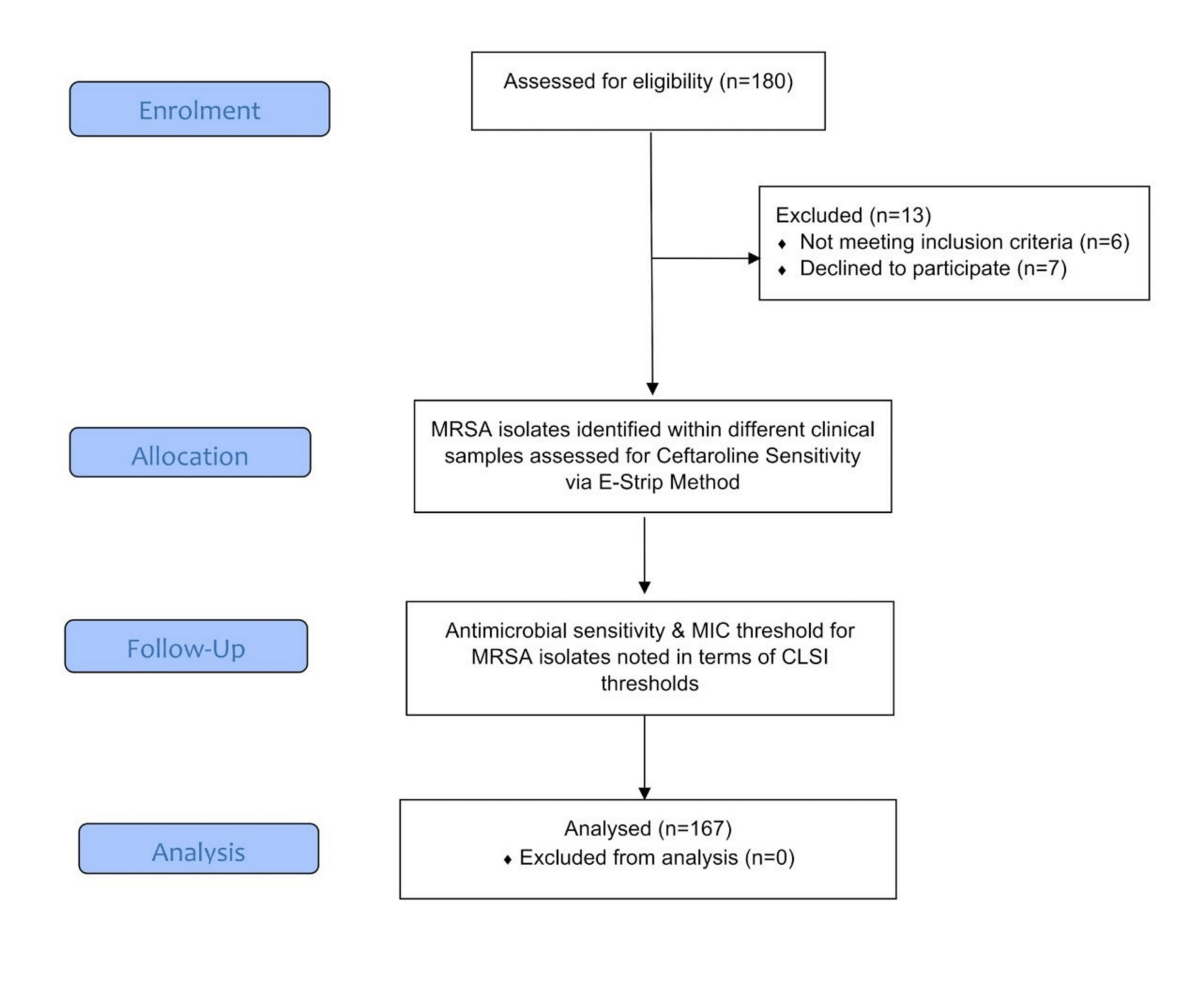
STROBE flow diagram CLSI, Clinical and Laboratory Standards Institute; MIC, minimum inhibitory concentration; MRSA, methicillin-resistant Staphylococcus aureus; STROBE, STrengthening the Reporting of OBservational studies in Epidemiology [10]

A total of 180 patients were initially assessed for eligibility during the study period. Of these, 13 patients were excluded, including six failing to meet the inclusion criteria and seven declining to participate. Finally, 167 MRSA isolates were included for the final analysis (Figure 1) [10].

Baseline patient characteristics

The study population had a mean age of 34.51 ± 21.35 years. Most participants were male, accounting for 103 (61.7%) of the cases. More than half of the isolates were obtained from patients in surgical departments (99; 59.3%), while the remaining (68; 40.7%) were from medical departments. Most patients were treated in hospital wards (134; 80.2%), followed by the OPD (26; 15.6%) and ICU (7; 4.2%) (Table 1).

**Table 1:** Baseline characteristics of included patients Data are presented as mean ± SD or number (percentage). DM, diabetes mellitus; SSTI, skin and soft tissue infection

Variables		Values
Age		34.51 ± 21.35
Gender	Male	103 (61.7)
Primary	Medical	68 (40.7)
	Surgical	99 (59.3)
Patient Site	Ward	134 (80.2)
	OPD	26 (15.6)
	ICU	7 (4.2)
Diagnosis	Diabetic foot	4 (2.4)
	SSTI	30 (18.0)
	Surgical site infection	61 (36.5)
	Catheter site infection	7 (4.2)
	Others	65 (38.9)
Type of specimen	Blood	26 (15.6)
	Sputum	10 (6.0)
	Tissue	25 (15.0)
	Urine	1 (0.6)
	Pus	90 (53.9)
	Others	15 (9.0)
Risk factors	All	50 (29.9)
	SSTI	16 (9.6)
	Uncontrolled DM	12 (7.2)
	Catheterization	16 (9.6)
	Immunosuppression	6 (3.6)

Clinical profile and specimen type

Surgical site infection was the most common diagnosis associated with MRSA isolates, accounting for 61 (36.5%) of cases. This was followed by skin and soft tissue infections (30; 18.0%). Other diagnoses included catheter site infections (7; 4.2%) and diabetic foot infections (4; 2.4%), while 65 (38.9%) were categorized under other conditions. In terms of specimen type, pus samples constituted the majority of MRSA isolates (90; 53.9%). Other specimen sources included blood (26; 15.6%), tissue (25; 15.0%), sputum (10; 6.0%), urine (1; 0.6%), and miscellaneous samples (15; 9.0%) (Table 1).

Risk factors

Overall, 50 (29.9%) of patients had identifiable risk factors. Among these, catheterization (16; 9.6%) and skin and soft tissue infection (16; 9.6%) were the most common. Uncontrolled diabetes mellitus was present in 12 (7.2%) patients, while immunosuppression was observed in six (3.6%) of cases.

Antimicrobial susceptibility pattern

**Figure 2:**
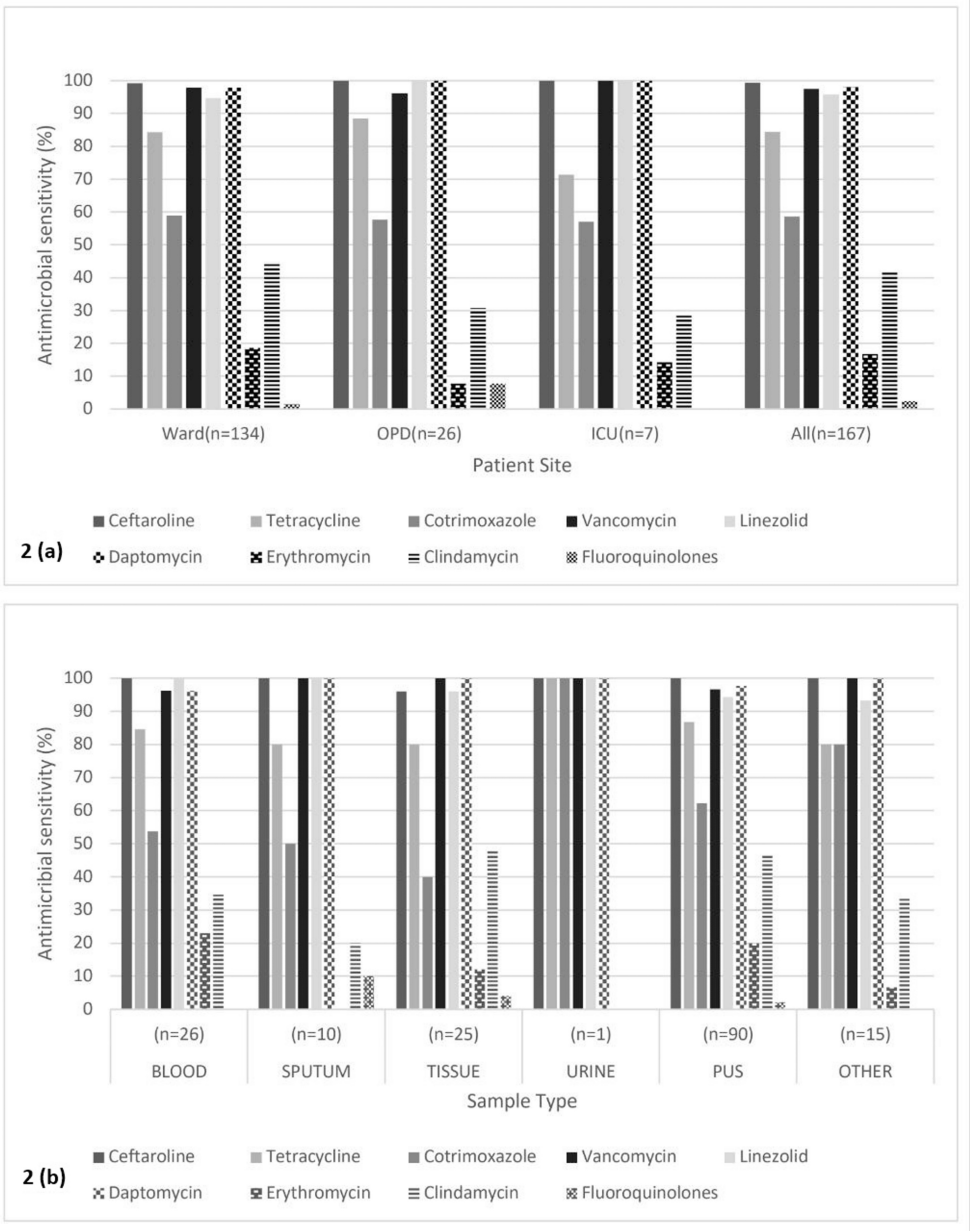
Antimicrobial susceptibility pattern based on patient site (a) and sample (b)

The antimicrobial susceptibility profile demonstrated high sensitivity rates for several antibiotics. Tetracycline, vancomycin, linezolid, and daptomycin showed sensitivity rates exceeding 80% among the MRSA isolates. However, some variation was observed across clinical settings. For example, tetracycline sensitivity was slightly lower in ICU isolates (approximately 70%) compared with other patient care areas (Figure 2a). Similarly, analysis by specimen type indicated that most sample categories maintained sensitivity rates above 80% for tetracycline, vancomycin, linezolid, and daptomycin. An exception was noted for urine isolates, although these demonstrated higher sensitivity to cotrimoxazole (Figure 2b).

Ceftaroline susceptibility and MIC distribution

**Figure 3:**
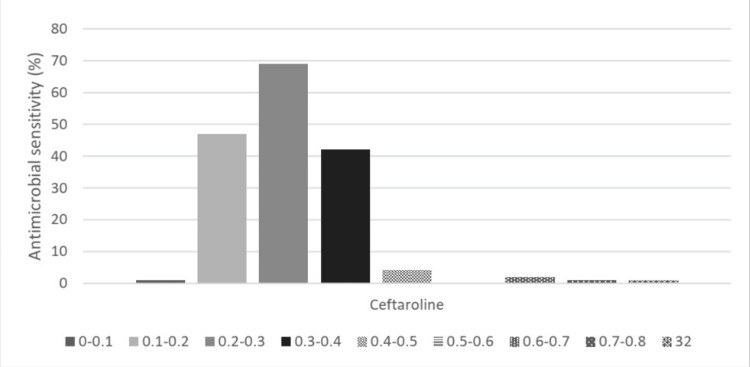
MIC values for ceftaroline sensitivity MIC, minimum inhibitory concentration

Among the 167 MRSA isolates, only one isolate demonstrated resistance to ceftaroline, with an MIC of 32 µg/mL. The remaining isolates exhibited MIC values predominantly ranging from 0.1 to 0.4 µg/mL, indicating a high degree of in vitro susceptibility to ceftaroline (Figure 3).

Overall, these findings demonstrate a very high susceptibility rate of MRSA isolates to ceftaroline, suggesting its potential effectiveness as a therapeutic option for MRSA infections in the studied tertiary care hospital setting.

## Discussion

The present prospective observational study evaluated the in vitro susceptibility of MRSA isolates to ceftaroline and analyzed antimicrobial susceptibility patterns in a tertiary care hospital setting. The findings demonstrate a very high susceptibility rate of MRSA isolates to ceftaroline, highlighting its potential role as an effective therapeutic option in the management of MRSA infections.

MRSA continues to be a major global public health concern because of its ability to develop resistance to multiple antibiotic classes and its association with significant morbidity and mortality [2,10]. Resistance to β-lactam antibiotics in MRSA occurs primarily due to the acquisition of the mecA gene, which encodes the altered PBP2a, resulting in reduced affinity for most β-lactam antibiotics [3]. Consequently, the treatment of MRSA infections often relies on alternative agents such as vancomycin, linezolid, and daptomycin, which remain the cornerstone of therapy in many clinical settings [11].

In the current study, 167 MRSA isolates were analyzed, with a mean patient age of 34.51 ± 21.35 years. A predominance of male patients (103; 61.7%) was observed, which is consistent with several hospital-based epidemiological studies reporting higher infection rates among males due to increased exposure to trauma, occupational risks, and surgical interventions [12]. Additionally, 99 (59.3%) of isolates were obtained from surgical departments, suggesting that surgical patients may be at greater risk of MRSA colonization or infection, particularly in the context of invasive procedures and wound exposure.

The clinical distribution of infections revealed that surgical site infections (61; 36.5%) were the most common diagnosis, followed by skin and soft tissue infections (30; 18.0%). This observation aligns with the well-established epidemiological pattern of MRSA infections, which frequently manifest as wound-related and soft tissue infections in both hospital and community settings [13]. Furthermore, the predominance of pus samples (90; 53.9%), as the primary source of MRSA isolates, supports the strong association between MRSA and wound-associated infections.

Risk factor analysis demonstrated that 50 (29.9%) of patients had identifiable risk factors, including catheterization (16; 9.6%), skin and soft tissue infections (16; 9.6%), uncontrolled diabetes mellitus (12; 7.2%), and immunosuppression (6; 3.6%). These findings are consistent with previous studies indicating that underlying comorbidities, invasive medical procedures, and impaired host immunity significantly increase the risk of MRSA infection [14].

The antimicrobial susceptibility analysis revealed high sensitivity rates (>80%) for tetracycline, vancomycin, linezolid, and daptomycin, which remain important therapeutic options for MRSA infections. However, minor variations were observed across patient care settings. For example, tetracycline susceptibility decreased to approximately 70% among ICU isolates, which may reflect increased antibiotic exposure and selection pressure in critically ill patients. Continuous antimicrobial surveillance is therefore essential to guide empirical therapy and limit the emergence of resistant strains.

The present study demonstrated that 166 of 167 MRSA isolates were susceptible to ceftaroline, corresponding to a susceptibility rate of 99.4%, while only one isolate (0.6%) showed resistance with an MIC of 32 µg/mL. Most isolates exhibited MIC values ranging from 0.1 to 0.4 µg/mL, indicating strong in vitro activity of ceftaroline against MRSA. These findings are consistent with several international studies reporting ceftaroline susceptibility rates exceeding 95% among MRSA isolates [4]. The effectiveness of ceftaroline can be attributed to its strong affinity for PBP2a, allowing it to overcome the methicillin resistance mechanism present in MRSA strains [15]. By targeting this altered protein, ceftaroline retains activity against strains that are resistant to most other β-lactam antibiotics. Ceftaroline has also demonstrated favorable clinical outcomes in patients with complicated skin and soft tissue infections and community-acquired pneumonia, including infections caused by MRSA [12]. In addition to its bactericidal activity, ceftaroline exhibits good tissue penetration and a favorable safety profile, which may make it a useful alternative in patients who cannot tolerate other anti-MRSA agents [16].

This study has several important strengths. First, it was conducted as a prospective observational study, allowing systematic collection of clinical and microbiological data. Second, a relatively large number of MRSA isolates (n = 167) from diverse clinical samples were analyzed, which improves the reliability of the findings. Third, the use of the E-test strip method for determining MICs provided precise quantitative susceptibility data for ceftaroline. Finally, the study evaluated both antimicrobial susceptibility patterns and clinical characteristics of patients, offering a comprehensive overview of MRSA epidemiology in a tertiary care setting.

Despite the encouraging findings, certain limitations should be acknowledged. The study was conducted in a single tertiary care center, which may limit the generalizability of the results to other healthcare settings. In addition, the study focused on in vitro susceptibility patterns, and clinical outcomes associated with ceftaroline therapy were not evaluated due to its unavailability. Our objective was to perform inferential analysis, but in the presence of only one ceftaroline-resistant isolate for comparison, we could not perform it. The relatively small number of ICU and urine samples may also have limited the ability to perform subgroup analyses. Larger multicenter studies with clinical outcome assessment would be valuable to further validate these results.

## Conclusions

The present study demonstrates excellent in vitro activity of ceftaroline against MRSA isolates, with a susceptibility rate exceeding 99%, and supports further multicenter studies incorporating clinical outcome data to evaluate its therapeutic role in tertiary care settings. However, its use should preferably be reserved for patients with MRSA infections resistant to other available anti-MRSA agents, based on antimicrobial susceptibility testing.
